# Life-Threatening Radiation Necrosis After Complete Response to Pembrolizumab in a Patient With a Metastatic Silent PIT1 Pituitary Neuroendocrine Tumor (PitNET)

**DOI:** 10.1097/CJI.0000000000000601

**Published:** 2026-02-23

**Authors:** Dario De Alcubierre, Tiziana Feola, Francesca Gianno, Monica Verrico, Francesca Carbonara, Eugenio Venditti, Andrea M. Isidori, Vincenzo Esposito, Giuseppe Minniti, Marie‐Lise Jaffrain-Rea

**Affiliations:** * Neuromed IRCCS, Pozzilli, Italy; † Department of Experimental Medicine, Sapienza University, Rome, Italy; ‡ Department of Radiological, Oncological and Pathological Sciences, Sapienza University, Rome, Italy; § Department of Biotechnological and Applied Clinical Sciences, University of L’Aquila, L’Aquila, Italy; ∥ Centre for Rare Diseases (ENDO-ERN Accredited), Policlinico Umberto I, Rome, Italy; ¶ Department of Neurology and Psychiatry, Sapienza University, Rome, Italy

**Keywords:** pituitary carcinoma, pituitary neuroendocrine tumor (PitNET), metastatic PitNET, PIT1, PD-L1, immune checkpoint inhibitors, radiation necrosis, pembrolizumab, pseudoprogression

## Abstract

Metastatic pituitary neuroendocrine tumors (metPitNETs) are rare neoplasms with limited therapeutic options. Temozolomide is the first-line therapy, but primary or secondary resistance frequently occurs. Immune checkpoint inhibitors (ICIs) are emerging as a promising second-line option; however, clinical experience remains limited. We report the long-term follow-up of a 62-year-old male patient who received pembrolizumab (PBZ) treatment for a silent metPitNET derived from the PIT1 lineage after multiple surgical and radiation therapies and temozolomide failure. PBZ was proposed based on extensive PD‐L1 expression by tumor cells. A remarkable clinical, radiologic, and metabolic response was soon observed, progressively leading to complete disease remission after 21 months of treatment, with moderate immune-related adverse events. However, an unexpected rapid neurological deterioration occurred, due to the progression of a pseudotumoral temporal radionecrosis surrounded by an impressive vasogenic oedema, requiring emergency neurosurgery 7 weeks after PBZ withdrawal. The temporal mass had progressively developed on a previous small temporal metastasis treated through stereotactic radiosurgery, the corresponding area was hypometabolic at ^18^FDG PET-CT imaging, and histopathologic examination confirmed extensive radionecrosis and the absence of residual tumor cells. This is the first documented complete response to ICI in a PIT1-derived metPitNET. However, this remarkable response was complicated by the severe evolution of a brain radionecrosis, probably favoured by long-term PBZ. This case underscores the need for multidisciplinary expertise to differentiate treatment effects from neoplastic progression and to carefully follow-up the patients for potentially severe late treatment-related complications. It also questions the optimal duration of treatment in responsive cases.

Metastatic pituitary neuroendocrine tumors (metPitNETs)/pituitary carcinomas are very rare, accounting for about 0.1% of pituitary tumors.^1^ Like their benign counterparts, they are classified clinically according to hormone secretion (functioning/nonfunctioning PitNETs) and histologically according to immunostaining for pituitary hormones and/or transcription factors defining their lineage of origin—pituitary-specific transcription factor 1 (PIT1), T-box transcription factor (TPIT), and steroidogenic factor 1 (SF1). Extrapituitary dissemination of metPitNETs frequently involves the central nervous system, with brain and/or spinal metastases. Metastatic PitNETs share many biological features with their aggressive counterpart (agPitNETs), clinically defined by an unusually rapid growth despite optimized conventional treatment modalities (surgery, antisecretory drugs, and radiotherapy) and treated according to similar guidelines, including temozolomide (TMZ) as a first-line systemic therapy.^1^ TMZ is effective in about 35% of the cases and progression may occur due to primary or secondary resistance, requiring alternative therapeutic strategies.^1,2^ Over the last decade, immune checkpoint inhibitors (ICI) targeting PD1/PD‐L1 and CTLA-4 have emerged as a potential second-line of treatment, with favourable responses in over 40% of PitNETs refractory to TMZ.^3,4^ Despite limited experience with ICI in PitNETs, the safety profile appeared acceptable, with common immune-related adverse events (irAEs). An important issue following stereotactic radiosurgery (SRS) for brain metastasis is the risk of radionecrosis, a phenomenon characterized by damage extending to surrounding normal brain tissue, which may occur in up to 50% of treated lesions.^5^ Although most are asymptomatic, radionecrotic brain lesions may present as growing masses, making the differential diagnosis between neoplastic progression and treatment-related side effects essential for clinical management. Magnetic resonance imaging (MRI) may not be conclusive due to frequent overlapping features, functional/metabolic imaging can be useful, but surgery can be necessary to confirm the diagnosis and relieve symptoms.^6^

We describe a life-threatening pseudotumoral brain radionecrosis requiring emergency neurosurgery in a patient treated by pembrolizumab (PBZ) for a silent PIT1-positive metPitNET, refractory to repeated surgery, radiotherapy, and TMZ. Notably, this complication occurred in the first case of complete response (CR) to ICI observed in this peculiar PitNET phenotype. While supporting the role of immunotherapy in this context, this case underscores the need for multidisciplinary expertise and questions the optimal duration of treatment.

## CASE DESCRIPTION

**FIGURE 1:**
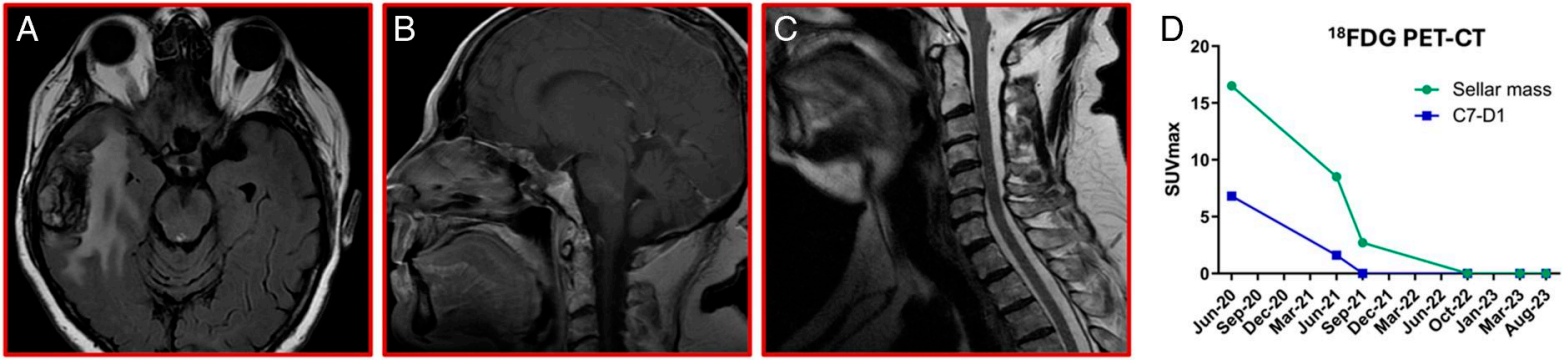
Complete radiologic and metabolic response to pembrolizumab. T1-weighted MRI performed after 21 months of treatment with pembrolizumab showing the absence of residual sellar disease—axial (A) and sagittal (B)—and the cervical spine (C). The temporal radionecrotic lesion surrounded by a noncompressive peripheral oedema was asymptomatic but already present (A). Evolution of the metabolic activity during ^18^FDG PET-CT imaging, as indicated by SUV measured on the primary sellar tumor (green line) and the spinal metastasis (blue line) (D). CT indicates computed tomography; FDG, fluorodeoxyglucose; PET, positron emission tomography; SUV, standardized uptake value. Adaptations are themselves works protected by copyright. So in order to publish this adaptation, authorization must be obtained both from the owner of the copyright in the original work and from the owner of copyright in the translation or adaptation Modified from Ref.^7^

The clinical history was partially reported as an illustrative case when the response to PBZ was still considered incomplete.^7^ Briefly, this male patient underwent transsphenoidal (TS) neurosurgery in November 2012 at the age of 57 years for a large, invasive, clinically nonfunctioning pituitary tumor, with a suprasellar extension determining headache and visual impairment. Complementary transcranial (TC) surgery was performed in June 2013, leading to subtotal tumor resection and visual normalization, with a small postoperative remnant in the left cavernous sinus. Both histopathologic reports concluded for a hormone-negative PitNET—formerly “null cell”—with unusual proliferative activity (Ki67 10%). The PIT1 lineage was identified subsequently, after the introduction of transcription factors immunostaining. One year later, he received stereotactic radiotherapy (STR) for an intrasellar/intracavernous tumor regrowth, with subsequent shrinkage and stabilization. In 2018, at the age of 62 years, he underwent a second TS approach for a sellar/infra-sellar recurrence presenting as obstructive nasal pseudopolyps. The increased proliferative potential of the tumor was confirmed histologically (Ki67 20%). Concurrently, 2 small prepontine nodules were noticed on MRI, reclassifying the tumor as a pituitary carcinoma/metPitNET. TMZ was started at 340 mg/day for 5 days every 28 days, along with complementary STR on the primary site and SRS on prepontine nodules. Despite an initial response, 2 small (<1  cm) asymptomatic right frontal and temporal nodules appeared, TMZ was shifted to a metronomic schedule (80  mg/d) and SRS was focally delivered in 2019. After a transient and asymptomatic disease stabilisation, spinal dissemination (C6-D1) appeared in 2020, together with a rapid infrasellar and anterior regrowth of the primary tumor causing nasal obstruction and visual loss in the right eye due to retrobulbar extension. Whole-body (WB) ^18^F-fluorodeoxyglucose (FDG) Positron Emission Tomography (PET)-Computed Tomography (CT) showed an intense hypermetabolic activity of recent intracranial and spinal lesions, but no residual uptake by irradiated brain metastases. Nonetheless, an asymptomatic right temporal heterogeneous nodule progressively appeared at MRI, radiologically consistent with radionecrosis. SRS was used for spinal metastasis and TMZ was permanently discontinued, while further molecular characterization of the primary tumor at the last surgery identified a strong, diffuse PD‐L1 immunopositivity (95% of tumor cells, SP263 clone-Ventana). Upon approval by the Ethics Committee, PBZ was started in off-label monotherapy (200  mg intravenously every 21  d) in March 2021. A rapid clinical response was observed, with visual and respiratory symptoms improving since the second cycle of treatment, resolving by the fourth cycle, when the radiologic shrinkage of sellar and spinal localisations was accompanied by a significant decrease in their metabolic activity. During the second year of treatment, a further sustained radiologic and metabolic improvement was observed. In November 2022, a large temporal hypometabolic area was observed at ^18^FDG PET-CT, in correspondence with the aforementioned nodule, which appeared enlarged (about 3  cm), haemorrhagic and surrounded by oedema in the white matter (Fig. 1). On the basis of the absence of neurological symptoms and combined imaging characteristics, radionecrosis was still considered as the first hypothesis and close follow-up was recommended. In May 2023, a complete radiologic and metabolic response to PBZ was documented, with a total disappearance of all brain, sellar, and spinal lesions at MRI—except for the temporal lesion, dimensionally stable—and the absence of metabolic activity at any previous tumor site on ^18^FDG PET-CT, with persisting hypometabolic characteristics of the right temporal area.

**FIGURE 2:**
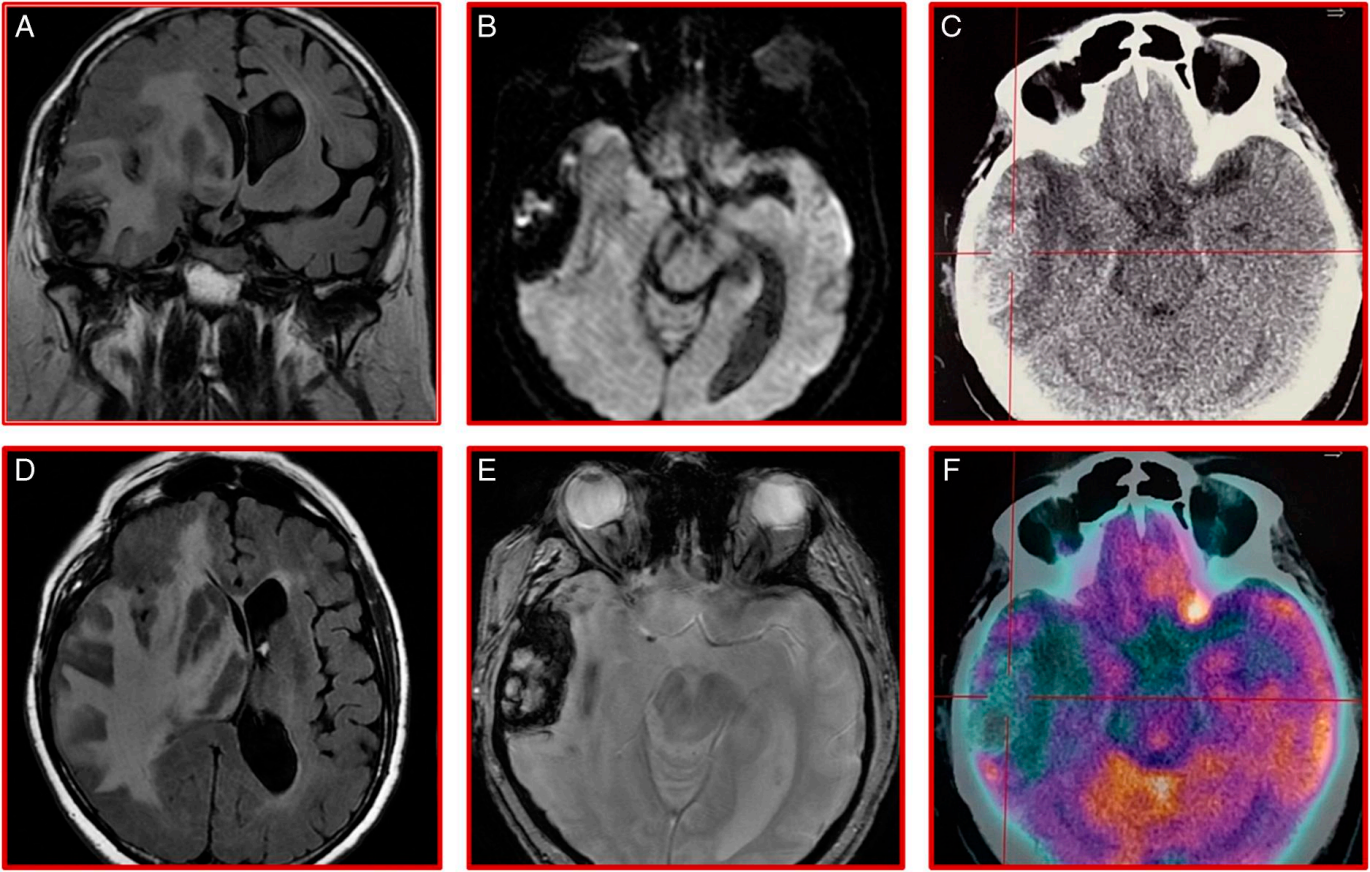
Right temporal radiation necrosis with significant perilesional oedema leading to midline shift. Multimodal neuroimaging of brain radiation necrosis. MRI FLAIR scan: the right temporal mass is shown on the coronal view (A), in which the sellar region appears tumor-free. The extensive perilesional oedema infiltrating the white matter, dissociating the basal nuclei and displacing the midline is also shown on the axial view (D). On axial DWI (B) and T1-weighted Gradient-Echo (E) views, the temporal mass is well-defined but heterogenous with necrotic areas, haemorrhage and hemosiderin deposits. The corresponding large hypometabolic area in the right temporal lobe is shown on 18FDG PET-CT scan (C,F).

**FIGURE 3:**
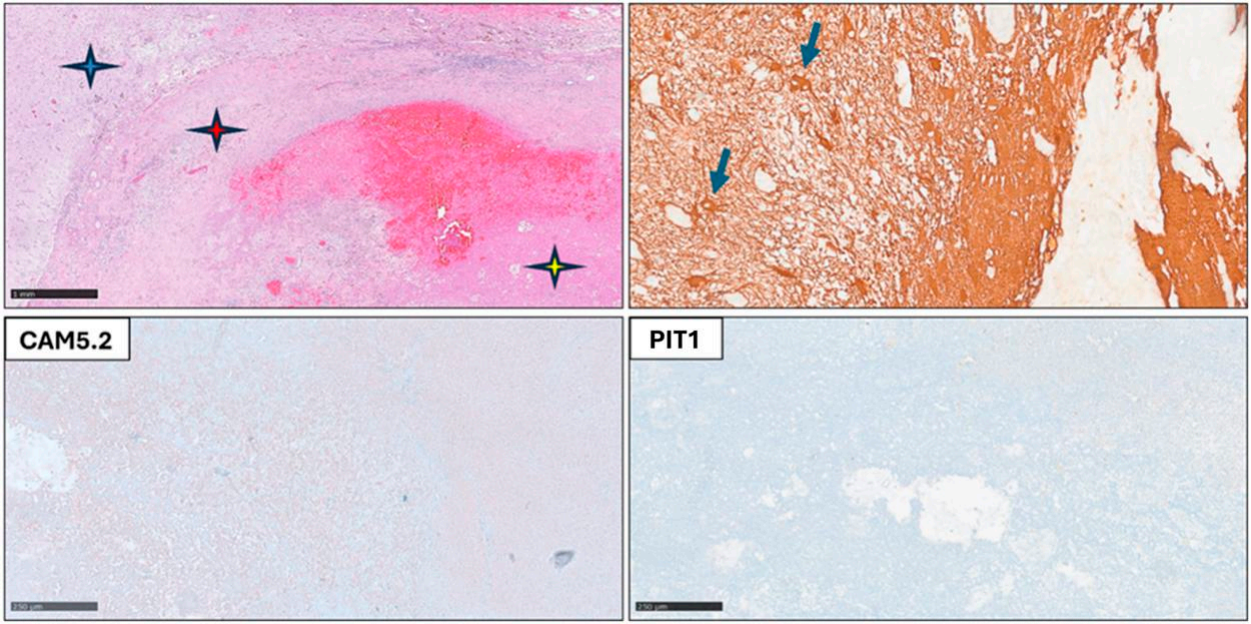
Histopathologic characteristics of the temporal radiation necrosis. Blue star: reactive brain parenchyma. Red star: fibrotic tissue with thick-walled vessels, macrophages, and lymphocytes. Yellow star: necrotic tissue. Positive staining for GFAP indicates reactive astrocytes (blue arrows) in the brain parenchyma. Negative staining for CAM5.2 and PIT1 confirms the absence of tumor cells.

As previously reported,^7^ PBZ treatment was complicated by some irAEs: (1) an early maculopapular skin rash (G1-G2), successfully managed with long-term low-dose systemic glucocorticoids (GC); (2) at the eighth cycle, renal dysfunction with significant hypertension (G3) prompted transient ICI withdrawal, but PBZ could be reintroduced at a maintenance dose. However, in March 2023, the patient started complaining of mild gait disturbances, which progressively worsened despite ongoing GC, leading to permanent discontinuation of PBZ in July 2023 after 29 cycles of treatment. Nonetheless, neurological symptoms rapidly worsened in August 2023 with ideomotor slowing, poor trunk control, and urinary incontinence. A dramatic progression of the right temporal area causing significant midline shift (Fig. 2) required emergency TC surgery on September 8th, 2023. No other disease localization was found. Histopathologic examination (Fig. 3) confirmed the presence of necrotic areas. The adjacent brain parenchyma exhibited reactive gliosis, abundant lymphoplasmacytic and macrophage infiltration, as well as widespread haemorrhagic extravasations with hemosiderin deposits. Stromal fibrosis and hyalinosis of vessel walls were also observed. Negative immunostaining for CAM5.2 and PIT1 confirmed the absence of residual tumor cells. Postoperative imaging documented the complete resection of the temporal lesion and regression of the surrounding oedema. Unfortunately, despite an immediate postoperative neurological improvement, gait disturbances of uncertain pathogenesis went on. Two months later, the patient was admitted twice at the local hospital because of feverish respiratory distress and urinary infection and died on November 25th, 2023, in a context of septic shock.

## DISCUSSION

Despite the established efficacy of ICI in several cancer types, <30 cases of ag/met PitNETs treated by mono or dual ICI have been reported, with a favourable outcome in nearly 35%, but few CR.^3,7^ To the best of our knowledge, this is the first CR achieved in a silent PIT1 metPitNET, the previous cases consisting of 2 metastatic (one lactotroph, one corticotroph) and one aggressive corticotroph PitNETs.^7^ It is interesting to note that metPitNETs appear more likely to respond to ICI than agPitNETs.^4^ Other potential predictors of response are unclear. Neither the histologic subtype nor the hormonal status of PitNETs appears to affect treatment outcome. As in our patient, a high PD‐L1 expression was associated with a better response in PIT1/lactotroph PitNETs, whereas a high tumor mutation burden and/or mismatch repair deficiency might be predictive in TPIT/corticotroph PitNETs.^3,7^ This should be further clarified. Herein, we wish to highlight the late and severe evolution of a brain radionecrosis developing after SRS given to a small temporal metastatic lesion, leading to unexpected rapid mass effects and requiring emergency surgery 7 weeks after PBZ withdrawal. This observation raises relevant issues, including the differential diagnosis of expanding masses developing on ICI and the optimal duration of treatment.

Atypical response patterns have been well-characterized on ICI, including “pseudoprogression,” “hyperprogression,” and dissociated responses, which should be correctly interpreted—especially during the first cycles of treatment—to appropriately continue the treatment if pseudoprogression occurs due to inflammation, or promptly withdraw ICI in case of hyperprogression. To this aim, modified RECIST criteria and iPERCIST criteria should be considered. In contrast, ICI may exacerbate pre-existing radiation necrosis by enhancing tumor immunogenicity and local inflammation.^8^ Radionecrosis is reported in up to 50% of irradiated brain metastasis, only 7%–16% becoming symptomatic,^9^ but ICI may increase this risk by 2.5 to 4-folds.^10^ The differential diagnosis between tumor progression and radionecrosis may be challenging. Both may appear on follow-up imaging or revealed by neurological symptoms, presenting as contrast-enhanced lesions surrounded by oedema, and if MRI (including perfusion studies) or functional imaging may be helpful, surgery may be necessary to achieve a definitive diagnosis.^6^ In our patient, surgery was clinically indicated and histopathologic examination confirmed the diagnosis of extensive radiation necrosis, already suspected on the basis of: (1) MRI showing a well-delimited heterogeneous temporal nodule arising in a previous field of irradiation, surrounded by oedema involving preferably the white matter, including arcuate fibres, (2) a clearly hypometabolic status on ^18^FDG PET-CT. Surgical resection is considered in the most severe cases, whereas GC or antiangiogenic therapy with bevacizumab (BVZ) are medical options in less symptomatic cases. In a retrospective evaluation of 217 patients who received SRS for brain metastasis, 26 developed symptomatic radionecrosis: GC induced complete and partial responses in 50% and 33% of the cases, respectively, with similar data for BVZ and a complete response in all operated cases.^11^ In our patient, radionecrosis and surrounding oedema slowly progressed on continuous low-dose GC for irAEs and rapidly worsened despite PBZ withdrawal. Histopathologic examination also confirmed the absence of residual tumor cells.

The occurrence of irAEs has been associated with a better response to ICI in several neoplasia.^12^ Our patient developed persistent skin eczema and acute nephritis, which were successfully managed with GC and transient drug discontinuation. However, gait disturbances of uncertain origin developed at the end of the second year of treatment. The persistence of symptoms together with CR led to permanent PBZ discontinuation. Central and peripheral neurological irAEs are relatively infrequent—1-6%, up to 13% on dual ICI—with variable clinical presentations and timing, sometimes occurring months after drug withdrawal, with a possible auto-immune pathogenesis supported by reports of neural autoantibodies.^13^ In our patient, iatrogenic neurotoxicity was deemed likely, but the maintenance of low-dose GC after PBZ withdrawal failed to prevent a late acute neurological degradation, attributed to radionecrosis. Noteworthy, while acute symptoms resolved post-operatively, gait disturbances of unclear origin persisted.

## CONCLUSION

The risk of severe, potentially life-threatening, adverse effects of ICI should be carefully balanced with drug efficacy in determining the optimal treatment duration, which is currently debated. In responsive cases, a minimum duration of 2 years is generally accepted if the tolerance is acceptable and recent studies suggest that the risk of recurrence is reduced by longer treatments.^14^ In ag/met PitNETs, PBZ or dual ipilimumab-nivolumab schedules have been used, PBZ alone being occasionally given for more than 2 years.^7^ We suggest carefully weighting the risk of late or delayed irAEs and/or symptomatic radionecrosis before extending treatment duration well beyond CR, especially in patients with metPitNET disseminating to the central nervous system who have received repeated brain irradiation, before or during TMZ, or during transition from an aggressive to metastatic disease.^2^

## CONFLICTS OF INTEREST/FINANCIAL DISCLOSURES

*Conflicts of interest: None reported. All authors have declared that there are no financial conflicts of interest with regard to this work.*

*Ethics approval: The off-label use of pembrolizumab in this patient was approved by the Ethics Committee.*

*Informed consent: The patient and his family provided written consent for the scientific use and anonymous publication of the clinical story, including any pertinent data and imaging, as well as immunohistochemical and molecular studies performed for diagnostic or research purposes.*

*Data availability statement: Anonymized original data and illustrations concerning the patient are available upon reasonable request to the corresponding author.*
